# Psychosocial Work-Related Hazards and Their Relationship to the Quality of Life of Nurses—A Cross-Sectional Study

**DOI:** 10.3390/ijerph17030755

**Published:** 2020-01-24

**Authors:** Bianka Misiak, Regina Sierżantowicz, Elżbieta Krajewska-Kułak, Karolina Lewko, Joanna Chilińska, Jolanta Lewko

**Affiliations:** 1 Medical University of Bialystok Children’s Clinical Hospital, 15-274 Bialystok, Poland; bianka.misiak@o2.pl; 2 Department of Surgical Nursing, Medical University of Bialystok, 15-086 Bialystok, Poland; renatasierz@wp.pl; 3 Department of Integrated Medical Care, Faculty of Health Sciences, Medical University of Bialystok, Marii Sklodowskiej-Curie 7a str, 15-096 Bialystok, Poland; elzbieta.krajewska@wp.pl; 4 International Medical Students Assossiation-Poland (IFMSA-Poland), Medical University of Bialystok, 15-089 Bialystok, Poland; karolinka6694@gmail.com; 5 Faculty of Health Science, Łomża State University of Applied Sciences, 18-400 Łomża, Poland; joasia.chilinska@op.pl

**Keywords:** work, nurses, health

## Abstract

Background: Nursing requires a commitment to work and care for the well-being of the patient, which is a great mental and physical burden for the nurse. As a result of exposure to adverse psychosocial work conditions and experiencing the resulting work-related stress, the problem of burnout is becoming more common. The aim of the study was to assess the psychosocial work conditions and their relationship to quality of life in the studied group of nurses. Methods: A cross-sectional study was carried out on 523 randomly selected professionally active registered nurses. The study was based on a diagnostic survey using standardized psychometric questionnaires: The Psychosocial Working Conditions Questionnaire and the quality of life WHOQOL-Bref. Results: Respondents with a better education assessed the level of demands at work to be higher (*p* = 0.000); however, they were also more satisfied in the well-being category (*p* = 0.020). Shift work was associated with a worse perception of psychosocial work conditions in almost all considered domains. The strongest correlations were between the scale of well-being and the assessment of quality of life in the somatic and psychological domains. Conclusion: Nurses doing shift work assessed working conditions as being worse in all domains. They felt the mental and physical burden the most. Psychosocial work conditions were assessed to be better by nurses working in management positions. The strongest correlations were between the scale of well-being and the assessment of quality of life in the somatic and psychological domains.

## 1. Introduction

Psychosocial hazards are naturally connected with experiencing work-related stress. Work-related stress, as a reaction of the human body, can occur in a situation when an individual is exposed to work-related demands and pressures that are not adapted to one’s knowledge and skills and that pose a challenge from the point of view of one’s ability to cope [1]. In addition, as a result of exposure to adverse psychosocial working conditions and experiencing the resulting work-related stress, the problem of burnout is also becoming more common. The incidence of burnout and its recognition has increased substantially over the last few years and has become a synonym for psychosomatic, psychological symptoms and social consequences of a long-lasting workload exceeding an individual’s capacity. In health services work, these dimensions relate to working with people who are a feature of this type of work and that manifest exhaustion resulting from interpersonal tensions (emotional exhaustion) and withdrawal from contact with service recipients (depersonalization) [1]. Nursing is a profession in which close, intense contact with other people plays a very integral role. A commitment to work and care for the well-being of the patient is a great mental and physical burden for the nurse [2]. An imbalance between the costs of work and the obtained profits may result in exhausting both the short-term energy resources, in the form of acute fatigue, and chronic energy resources, in the form of burnout [3]. Occupational burnout can be defined as physical, mental and emotional exhaustion caused by long-term involvement in situations related to emotional burden [2]. To obtain information on the impact of the environment or work organization on human health and the quality of the work performed, and vice versa—the human on the environment/organization—it is necessary to collect data on both the human and the environment/organization [4]. Earlier studies conducted in a group of Polish nurses by Kowalczuk and Krajewska-Kułak [5] show that age, duration of employment at the present institution and at a current position correlated significantly with the scores of the demands and desired changes scales as well as with the values of the psychophysical demands subscale.

The aim of the study was to assess the psychosocial work conditions and their relationship to the quality of life in the studied group of nurses with many years of experience in the profession.

## 2. Materials and Methods

### 2.1. Design and Participants

A cross-sectional study was carried out on a group of consecutively, randomly selected and professionally active registered nurses from an eastern voivodeship in Poland, aged over 40 years old, who agreed to participate in the study and filled out a survey. The study design and reporting format are in accordance with the recommended Strengthening the Reporting of Observational Studies in Epidemiology guidelines for observational clinical studies. The inclusion criteria were (1) individuals aged 40 years old or above, and (2) individuals working as nurses. Nurses working in hospitals, various acute ward types, outpatient specialist care, primary health care, and others who agreed to participate were included in the study.

A total of 560 questionnaires were distributed with the collaboration of the Chamber of Nurses and Midwives, and 523 envelopes were collected; they were the basis for the empirical analysis. The study protocol underwent several approval procedures by the bioethics commission and the administrations of the institutions where the nurses worked.

The collected material was encoded, saved in an Excel spreadsheet, and subjected to verification. Only completed questionnaires were included in the statistical analysis.

The study was based on a diagnostic survey using standardized psychometric questionnaires: The Psychosocial Working Conditions Questionnaire [6], the quality of life WHOQOL-Bref [7], and an ad-hoc questionnaire comprised of questions pertaining to the social situation as well as information about the respondent.

The Psychosocial Working Conditions Questionnaire (PWCQ) is used to measure stress connected with psychosocial work characteristics. The tool enables measuring three variables distinguished in the concept of stress at work, which was developed by Robert Karasek [8,9]. These variables are demands, control, and social support. The most unfavorable from the point of view of stress is a situation where high demands are accompanied by low levels of control and social support. The PWCQ consists of five theoretical scales. They include, as per Cieślak and Widerszal-Bazyl [6], the scale of demands—what does your work demand? It includes intellectual, psychophysical, and safety responsibilities and requirements, resulting from role conflict and overload. It consists of 25 questions; the scale of control—to what extent can you influence what happens at work? It defines behavioral and cognitive control, consisting of 20 questions; the scale of social support—what kind of support can you count on? It assesses support from superiors and colleagues and consists of 16 questions; the scale of well-being—how do you feel? It assesses physical and mental well-being, consisting of 22 questions; and the scale of desired changes—do you expect any changes at work? The scale contains 20 closed and one open question. The PWCQ proved to be reliable, the Cronbach’s alpha index (coherence index) was adopted as a measure of reliability, as well as the so-called credibility estimate (two measurements using the same tool at a specified time interval). All theoretical scales are characterized by a high internal consistency (α from 0.82 to 0.94, depending on the scale) [6,10]. The PWCQ can be used for both individual and group studies. The studied person is required to mark one correct answer to each question based on a five-point Likert scale. The raw results are calculated according to the procedure given by the authors of the scale, thus obtaining the average results of the scale or subscales. The obtained values should be in a range from 1 to 5. The questionnaire has standards that were developed for eight professional groups, such as banking and insurance specialists, nurses, construction workers, salesmen, administration officials, IT specialists, public transport drivers, and teachers [6].

The quality of life WHOQOL-Bref questionnaire consists of 26 questions that enable assessing the quality of life profile in four domains: physical, psychological, social functioning, and functioning in the environment. The maximum number of points that can be obtained in each domain is 20 points; the higher the points, the better the quality of life. The calculations were done using the syntax WHOQOL-Bref program, and then the results were transformed into comparable results obtained with the full version of the quality of life WHOQOL-100, which enabled the results of different domains to be interpreted and compared using Cronbach’s alpha coefficients ranging from 0.92 to 0.94 [7]. The WHOQOL-Bref was adapted into the Polish language by Jaracz et al. [11].

### 2.2. Procedure and Ethical Considerations

The research conformed with good clinical practice guidelines, and the followed procedures were in accordance with the Helsinki declaration. The study was performed from January until December 2015. All nurses signed a consent form to participate in the study. The research was approved by the Bioethics committee of the Medical University of Bialystok (Resolution no. R-I-002/521/2014).

### 2.3. Statistical Analysis

During the study, we created a database (a source of variables) with nurses’ statements, opinions, and assessments, enabling the use of computational techniques. The analysis of each quantitative variable was conducted by calculating the mean (M), SD, median (Me), lower (Q1) and upper quartile (Q3), and minimum (Min) and maximum (Max) values. Relationships between selected factors and psychometric measures were analyzed. Depending on the variables being compared, the chi-square test for independence and Spearman’s rank correlation analysis were used. The Mann–Whitney test and the Kruskal–Wallis test were used to assess the significance of the differences between the groups. Study results of *p* < 0.05 were regarded as statistically significant.

## 3. Results

### 3.1. Characteristics of the Studied Group

Two-thirds of the nurses worked over 20 years in the profession, and the vast majority (79%) did shift work. The shift work lasted 12 h a day and 12 h a night and there were no nurses working only at night. Every tenth studied nurse (11%) had a managerial position. Among the studied nurses, the largest group included people aged 41–45 years old, and the next was those between 46 and 50. A total of 19% of the respondents were in the range of 51–55 years, and 12% were between 56 and 60. Almost a quarter of the respondents (23%) had a Master’s degree, 36% had a Bachelor’s, and 38% had a secondary-level education. Half of the respondents were employed in one place, almost 30% had two places of employment, and 16% of the group worked at more than two places.

### 3.2. Factors Affecting Assessment of Working Conditions

Age only differentiated the assessment of work demands set for the studied nurses (*p* = 0.005); slightly higher values were observed in women up to 50 years old in this category. These demands concerned intellectual and psychophysical demands as well as responsibility for their own patient and safety.

Education was a significant factor affecting the assessment of psychosocial work conditions. The best educated respondents assessed the level of demands at work as higher (*p* = 0.000); however, they were also more satisfied in the well-being category (*p* = 0.020). In terms of education, the need for change was noticed by nurses after completing their undergraduate studies (*p* = 0.000). People with a secondary education did not see the need to change the work conditions.

Work experience also differentiated the assessments of work conditions, although the direction of these correlations was not entirely clear. For example, when it comes to assessing the need for change, the highest average was obtained in the group of nurses working for 16–20 years (*p* = 0.001).

The financial situation affected the assessment of work conditions very strongly. Here, the correlations were observed and it was concluded that people in a good financial situation assessed certain aspects of work better than people in worse financial situations. The data are presented in Table 1.

People with a specialization rated the demands placed on a nurse to be higher. Shift work was associated with varying assessments of psychosocial work conditions in almost all the considered domains. People doing shift work had a lower assessment of the possibility of self-control on the range of activities or nursing interventions performed at work (*p* = 0.000), felt less social support (*p* = 0.015), and saw more of a need to change work conditions (*p* = 0.026).

Work conditions were better assessed by people in management positions as opposed to other nurses. The more places of employment of the nurses, the higher the assessment of the demands placed on a nurse. Other aspects of psychosocial work conditions were assessed similarly, regardless of the number of jobs. With the exception of the scale of demands, all measures assessing psychosocial work conditions were associated with an overall sense of job satisfaction and were statistically highly significant (test probability value of *p* < 0.001). The higher the overall job satisfaction, the higher the sense of control over the carried-out activities and the greater the social support and overall scale of well-being. As well, the feeling of needing to make changes at work was lower.

The correlations of satisfaction with financial remuneration with the assessment of psychosocial work conditions were of a similar nature, except that the assessment of the level of demands also depended on satisfaction with the salary received (Table 2).

Using the questionnaire to assess psychosocial work conditions, five aspects of nurses’ work were assessed: the demands that work places on the employee, control over the work carried out, social support during work performance, general well-being, and expectations of changes in work. The scores for each category range from 1 to 5 points. Table 3 presents a description of the distribution of the five calculated measures, as well as their subscales. Higher values in the results of the scale of demands and desired changes were evidence of worse working conditions. The remaining three measures should have the highest possible values, as that indicates that the employee controls the scope of his/her duties better, has a greater sense of social support, and an overall better quality of life and carrying out of work.

A rather negative picture of the nurses’ work conditions emerged. The measures of demands (M = 3.54; CI 95% = 3.10–3.58) and the need for changes (M = 3.62; CI 95% = 3.51–3.74) had high values. The assessments of the positive aspects of work were much lower, except for well-being. It is worth noting some detailed results; for example, when it comes to the scale of demands, the nurses felt much more burdened in the psychophysical aspect (M = 4.20; CI 95% = 3.98–4.25) than in the intellectual (M = 3.30; CI 95% = 3.10–3.58) (Table 3).

Measures of quality of life were also positively correlated with three aspects of assessing the psychosocial work conditions: scale of control, social support, and well-being. The strongest correlations were between the scale of well-being and the assessment of quality of life in the somatic and psychological domains (*R* exceeded 0.600). Other statistically significant correlations had little or no power. Detailed data are presented in Table 4.

## 4. Discussion

This research presents the psychosocial work conditions and their correlations with quality of life in a group of nurses. We decided to conduct the study in an older group of nurses, as the problem of the shortage of qualified medical staff in health care in Poland has been emphasized many times. In the case of nurses, we can talk of a crisis, manifested as an increase in the number of nurses in the highest age ranges, migrating, and leaving the profession, with a simultaneous increase in the demand for care and nursing services; the situation of the nurses’ environment can currently be described as alarming [12].

Based on the results of the conducted research, a rather critical picture of the work conditions in the nursing profession emerged. The measures of demands and the need for changes had high values. The assessment of the positive aspects of work were much lower, except for well-being. Some detailed results deserve attention; for example, when it comes to the scale of demands, nurses felt them much more in the physical than in the intellectual aspect.

The fulfillment of the tasks required of medical staff is often accompanied by physical and psychological fatigue. Therefore, the organization of health work consists of several activities that must be performed at a certain time with a certain number of nurses.

Other authors state that this is the result of 12 h of work, the high number of working hours, and insufficient rest between shifts [13].

The specificity of the nurse’s work in the patient’s environment causes a great physical burden. This is the result of nursing and rehabilitation activities, often performed in an inclined position, and the need to lift and move patients. In turn, the psychological burden is the result of often engaging in the health and life situation of the patients, as well as the conditions in which services are provided. At the same time, longer working times result from the need to move nurses between the patients’ places of residence, and there are burdens associated with carrying a bag with equipment necessary to provide nursing interventions [14].

In terms of changes at work, Kowalczuk et al. found that more experienced nurses, i.e., older and with longer work experience, were more critical of the work environment and felt the need for major changes at work. The respondents were also very pessimistic of conflicts in the workplace and being overburdened with responsibilities [15].

The results of other authors indicate that 52% of nurses in the emergency department in Ireland experienced high levels of emotional exhaustion and depersonalization, which were largely related to the nature of their work environment. To reduce work-related hazards, it is necessary to improve the environment and education [16].

In a meta-analysis, Zhang et al. found that, in nursing, the incidence of fatigue and emotional burnout is high. Better education and training can reduce fatigue and burnout and can improve the quality of life of nurses [17].

Work-related demands, especially physical demands, correlate with perceived health status. In the research of Habibi et al. [18] it was observed that psychosocial and ergonomic factors increase the intensity of lower-back discomfort of nurses in emergency units. Psychological work-related demands were associated with long-term absences and anticipated long-term poor health. Periodic health perception checks among active employees identify employees at increased risk. Using multiple regression, Watts et al. [19] showed that burnout can be predicted based on the organizational culture and there were significant associations between organizational culture and nurses’ sense of personal. Therefore, it seems that the perception of the work environment may affect the well-being of nurses.

Work-related demands, especially physical demands, correlated with perceived health status. Poor health predicted long-term disease-related absences. Early diagnosis of poor health should be the basis of a strategy to prevent long-term absences from work [20].

Nurses in Shanghai suffered from high levels of burnout, which was strongly associated with work-related stress. Interventions are necessary to reduce work-related stress, to reduce the burden of burnout among Chinese nurses [21].

In the conducted research, we found that education was a factor that significantly affected the assessment of work conditions. The best educated respondents assessed the level of demands at work higher and were more satisfied in the well-being category. People with a secondary education did not see the need to change work conditions.

According to research by Kowalczuk et al., the studied nurses rated the intellectual demands much lower than the psychophysical ones. Assessment of intellectual demands decreased with a lower level of education, whereas nurses working in management positions assessed intellectual demands higher. This assessment was not affected by the respondents’ work experience or age [15].

In the present study, with the exception of the scale of demands, all measures of psychosocial assessment of work conditions were correlated with the overall sense of job satisfaction and these correlations are statistically highly significant. The higher the overall job satisfaction, the higher the sense of control over the activities carried out and the greater the social support and overall scale of well-being, whereas the feeling of needing to make changes at work was lower. The work experience of the studied nurses also differentiated assessments of work conditions, although the direction of these correlations was not entirely clear. When assessing the need for changes, the highest average was obtained in the group of nurses with undergraduate degrees, whereas work conditions were better assessed by people in management positions.

We also found that people in management positions were not willing to change. According to Kowalczuk et al., this may be the result of their low involvement, because they have more opportunities than regular personnel to initiate changes at work [15].

The nurses’ financial situations affected the assessment of work conditions very strongly. Here, the correlations were logically oriented: Those in a good financial situation assessed certain aspects of work better than ones in worse situations.

The nurses’ financial situation significantly determined quality of life as well as mental health components, and a higher assessment of negative mental health symptoms pertained to nurses in worse financial situations [22].

In the conducted study, the strongest correlations were between the scale of physical and mental well-being and the assessment of quality of life in the somatic and psychological domains. Other correlations in the domains of social functioning and the environment of the WHOQOL-Brief already had little or quite negligible strength.

During screening, Wang et al. showed that burnout was the most important factor of poor quality of working life of nurses and can be important in assessing psychosocial work conditions to take measures in order to improve quality of life. [23].

In the studied nurses, shift work was associated with varying assessments of psychosocial work conditions in almost all the considered domains. People doing shift work assessed the possibility of self-control of activities performed at work lower, felt less social support from coworkers and management, and saw a greater need to change work conditions.

Low management support was an important prognostic factor for higher burnout and fatigue among emergency department nurses, while high levels of managerial support contributed to a higher level of satisfaction with compassion [24].

Kowalczuk et al. [15] tackled the issue of varying levels of social support in the workplace. Colleagues were more supportive than superiors. With age and work experience, the assessment of social support decreased, while it increased with education. In terms of well-being, the studied nurses rated their mental well-being higher than their physical conditions, regardless of their position. Nurses with a higher education assessed their well-being better, while with age and work experience, well-being assessments decreased.

Our study results show that the more places of employment there are, the higher the assessment of the demands placed on a nurse.

Shift work and having a second job reduced life satisfaction among oncology nurses [25].

Other aspects of psychosocial work conditions were assessed similarly, regardless of the number of jobs. However, shift work applies to most nurses employed in hospitals and the aim is to ensure continuity of care for patients. Working at night disturbs family and social functioning and negatively affects the employee’s health [5].

Our research suggests that the work conditions of the studied nurses need improvement, especially when it comes to support and reducing the physical and mental burden, particularly people working shifts.

There is a demand to provide clear rules, guidelines, and practical solutions for preventive care of employees in terms of psychosocial hazards. It is particularly recommended to share it with health care entities responsible for shaping safe and healthy work conditions [26].

## 5. Limitations of the Study

Our study was conducted only in one region of Poland, and the findings may not apply to other regions where working conditions can be viewed differently by nurses. Therefore, future studies with more diverse samples are required.

## 6. Conclusions

Nurses doing shift work assessed working conditions as worse in all domains. They felt the mental and physical burden the most. Psychosocial work conditions were assessed better by nurses working in management positions. The strongest correlations were between the scale of well-being and the assessment of quality of life in the somatic and psychological domains.

## Figures and Tables

**Table 1:** Selected socio-demographic factors and psychosocial working condition among respondents.

	Psychosocial Working Conditions				
Socio-Demographic Factors	Demands Scale	Control Scale	Social Support Scale	Well-Being Scale	Desired Changes Scale
	M ± SD, Me	M ± SD, Me	M ± SD, Me	M ± SD, Me	M ± SD, Me
**Age**					
41–45, *n* = 203	3.6 ± 0.4, 3.6	2.9 ± 0.4, 2.9	2.7 ± 0.8, 2.8	3.5 ± 0.5, 3.5	3.6 ± 0.6, 3.7
46–50, *n* = 153	3.5 ± 0.4, 3.6	3.0 ± 0.4, 3.0	2.9 ± 0.7, 2.9	3.4 ± 0.6, 3.4	3.6 ± 0.6, 3.7
51–55, *n* = 97	3.4 ± 0.4, 3.4	3.0 ± 0.4, 2.9	2.7 ± 0.7, 2.8	3.4 ± 0.6, 3.4	3.5 ± 0.7, 3.5
over 55, *n* = 62	3.5 ± 0.3, 3.5	2.9 ± 0.4, 2.9	2.7 ± 0.8, 2.8	3.4 ± 0.6, 3.5	3.4 ± 0.7, 3.5
*p* value	0.005	0.220	0.279	0.354	0.197
**Work experience (in years)**					
up to 15, *n* = 50	3.5 ± 0.3, 3.6	2.9 ± 0.4, 2.8	2.8 ± 0.7, 2.9	3.5 ± 0.5, 3.4	3.5 ± 0.7, 3.7
16–20, *n* = 128	3.6 ± 0.4, 3.6	2.9 ± 0.4, 2.9	2.6 ± 0.8, 2.7	3.4 ± 0.5, 3.5	3.7 ± 0.6, 3.7
21–25, *n* = 345	3.5 ± 0.4, 3.5	3.0 ± 0.4, 2.9	2.8 ± 0.8, 2.9	3.5 ± 0.6, 3.4	3.5 ± 0.6, 3.5
*p* value	0.001	0.016	0.114	0.876	0.020
**Education**					
Post-secondary, *n* = 200	3.4 ± 0.3, 3.4	2.9 ± 0.4, 2.9	2.8 ± 0.7, 2.9	3.4 ± 0.6, 3.4	3.5 ± 0.6, 3.5
Bachelor’s degree, *n* = 187	3.6 ± 0.4, 3.6	2.9 ± 0.4, 2.9	2.8 ± 0.8, 2.9	3.4 ± 0.5, 3.4	3.6 ± 0.7, 3.7
Master’s, *n* = 136	3.7 ± 0.3, 3.7	3.0 ± 0.5, 3.0	2.7 ± 0.9, 2.7	3.6 ± 0.5, 3.6	3.6 ± 0.7, 3.7
*p* value	0.000	0.554	0.349	0.020	0.038
**Financial situation**					
Good, *n* = 188	3.5 ± 0.4, 3.6	3.0 ± 0.5, 3.0	2.9 ± 0.9, 2.9	3.6 ± 0.5, 3.6	3.5 ± 0.7, 3.6
Average, *n* = 331	3.6 ± 0.4, 3.6	2.9 ± 0.4, 2.9	2.7 ± 0.7, 2.8	3.4 ± 0.5, 3.4	3.6 ± 0.6, 3.6
Bad, *n* = 14	3.4 ± 0.5, 3.5	2.8 ± 0.4, 2.9	2.4 ± 0.8, 2.4	3.1 ± 0.6, 3.1	3.6 ± 0.6, 3.7
*p* value	0.363	0.009	0.014	0.000	0.649

**Table 2:** Selected professional and personal factors and respondents’ psychosocial working conditions.

	Psychosocial Working Conditions				
Professional Factors	Demands Scale	Control Scale	Social Support Scale	Well-Being Scale	Desired Changes Scale
	M ± SD, Me	M ± SD, Me	M ± SD, Me	M ± SD, Me	M ± SD, Me
**Shift work**					
Yes, *n* = 413	3.5 ± 0.4, 3.6	2.9 ± 0.4, 2.9	2.7 ± 0.8, 2.8	3.4 ± 0.5, 3.4	3.6 ± 0.6, 3.6
No, *n* = 110	3.5 ± 0.4, 3.5	3.1 ± 0.4, 3.1	2.9 ± 0.7, 3.0	3.5 ± 0.5, 3.4	3.4 ± 0.7, 3.5
*p* value	0.053	0.000	0.015	0.470	0.026
**Work in a managerial position**					
Yes, *n* = 56	3.7 ± 0.4, 3.6	3.2 ± 0.5, 3.2	2.9 ± 0.8, 3.0	3.5 ± 0.5, 3.4	3.4 ± 0.7, 3.5
No, *n* = 467	3.5 ± 0.4, 3.5	2.9 ± 0.4, 2.9	2.7 ± 0.8, 2.8	3.5 ± 0.5, 3.4	3.6 ± 0.6, 3.6
*p* value	0.002	0.000	0.712	0.721	0.090
**Number jobs**					
One, *n* = 289	3.5 ± 0.4, 3.5	2.9 ± 0.4, 2.9	2.8 ± 0.8, 2.9	3.5 ± 0.6, 3.4	3.6 ± 0.6, 3.6
Two, *n* = 148	3.5 ± 0.4, 3.5	3.0 ± 0.4, 2.9	2.7 ± 0.7, 2.8	3.5 ± 0.5, 3.5	3.5 ± 0.7, 3.6
More, *n* = 86	3.7 ± 0.4, 3.7	3.0 ± 0.5, 3.0	2.7 ± 0.7, 2.8	3.4 ± 0.5, 3.4	3.6 ± 0.6, 3.6
*p* value	0.000	0.668	0.480	0.647	0.553
**Specialization in nursing**					
No, *n* = 290	3.5 ± 0.4, 3.5	2.9 ± 0.5, 2.9	2.7 ± 0.8, 2.8	3.4 ± 0.6, 3.4	3.6 ± 0.6, 3.6
Yes, *n* = 233	3.6 ± 0.4, 3.6	3.0 ± 0.4, 3.0	2.8 ± 0.8, 2.9	3.5 ± 0.5, 3.5	3.5 ± 0.7, 3.6
*p* value	0.049	0.270	0.260	0.082	0.777
**Satisfaction with financial remuneration**					
Low	3.6 ± 0.4, 3.6	2.9 ± 0.4, 2.9	2.7 ± 0.8, 2.7	3.4 ± 0.5, 3.4	3.6 ± 0.6, 3.6
Average	3.4 ± 0.4, 3.4	3.1 ± 0.4, 3.0	3.0 ± 0.6, 3.0	3.5 ± 0.5, 3.5	3.5 ± 0.6, 3.6
High	3.4 ± 0.3, 3.3	3.2 ± 0.4, 3.2	3.1 ± 0.9, 3.0	3.7 ± 0.6, 3.7	3.2 ± 0.8, 3.1
*p* value	0.000	0.000	0.000	0.011	0.020
**Feeling of job satisfaction**					
Low	3.6 ± 0.5, 3.6	2.7 ± 0.4, 2.7	2.3 ± 0.8, 2.2	3.0 ± 0.6, 3.1	3.8 ± 0.6, 3.8
Average	3.5 ± 0.4, 3.5	2.9 ± 0.4, 2.8	2.6 ± 0.7, 2.7	3.4 ± 0.5, 3.4	3.7 ± 0.6, 3.7
High	3.5 ± 0.4, 3.5	3.1 ± 0.4, 3.1	3.0 ± 0.5, 3.6	3.6 ± 0.5, 3.6	3.4 ± 0.7, 3.4
*p* value	0.507	0.000	0.000	0.000	0.000

**Table 3:** Average values of the Psychosocial Working Conditions subscales and WHOQoL-BREF parameters among respondents.

	M	Me	*SD*	*c*_25_	*c*_75_	min.	max.
Psychosocial Working Conditions							
Demands Scale ↓	3.54	3.56	0.39	3.24	3.80	2.24	4.48
CI 95% (3.10–3.58)							
—intellectual demands	3.30	3.22	0.54	3.00	3.67	1.67	4.78
CI 95% (3.05–3.48)							
—psychophysical	4.20	4.22	0.48	3.89	4.56	2.11	5.00
CI 95% (3.98–4.25)							
—resulting from responsibility for safety	2.93	2.83	0.61	2.50	3.33	1.33	4.83
CI 95% (2.78–2.99)							
Control Scale ↑	2.96	2.95	0.43	2.70	3.20	1.70	4.55
CI 95% (2.78–3.07)							
—behavioral control	2.37	2.30	0.57	1.90	2.80	1.00	4.80
CI 95% (2.29–2.46)							
—cognitive control	3.60	3.56	0.57	3.11	4.00	2.11	5.00
CI 95% (3.37–3.72)							
Social Support Scale ↑	2.76	2.88	0.78	2.25	3.31	1.00	5.00
CI 95% (2.66–2.84)							
—support from superiors	2.58	2.63	0.90	1.88	3.13	1.00	5.00
CI 95% (2.41–2.66)							
—support from co-workers	2.95	3.00	0.82	2.50	3.50	1.00	5.00
CI 95% (2.69–3.15)							
Well-Being Scale ↑	3.46	3.45	0.55	3.14	3.82	1.77	5.00
CI 95% (3.33–3.59)							
—physical well-being	3.56	3.55	0.63	3.18	4.00	1.64	5.00
CI 95% (3.39–3.71)							
—mental well-being	3.37	3.36	0.55	3.09	3.73	1.82	5.00
CI 95% (3.26–3.45)							
Desired Changes Scale ↓	3.56	3.60	0.65	3.10	4.00	1.25	4.95
CI 95% (3.29–3.64)							
—need for change	3.62	3.68	0.68	3.16	4.05	1.26	5.00
CI 95% (3.51–3.74)							
**WHOQoL-BREF**							
Somatic domain	12.9	13.1	1.9	12.0	14.3	4.6	17.7
CI 95% (12.7–13.1)							
Psychological domain	13.7	14.0	1.9	12.7	14.7	5.3	14.7
CI 95% (13.5–13.9)							
Social domain	14.6	14.7	2.9	13.3	16.0	4.0	20.0
CI 95% (14.3–14.8)							
Environment	13.2	13.5	2.2	12.0	14.5	5.0	19.0
CI 95% (13.0–13.4)							

CI95%—confidence interval, ↓—the scales are negative (higher values mean worse working conditions), ↑—scales are positive (higher values means better working conditions).

**Table 4:** Spearman’s rank correlation coefficient between quality of life and respondents’ psychosocial working conditions.

Psychosocial Working Conditions	Quality of Life WHOQOL-Bref			
	Somatic Domain	Psychological Domain	Social Domain	Environment
Demands Scale	−0.05	0.00	0.06	−0.06
	*p* = 0.278	*p* = 0.0000	*p* = 0.1858	*p* = 0.144
Control Scale	0.24	0.28	0.16	0.33
	*p* = 0.0000	*p* = 0.0000	*p* = 0.0000	*p* = 0.0000
Social Support Scale	0.17	0.16	0.21	0.20
	*p* = 0.0000	*p* = 0.0000	*p* = 0.0000	*p* = 0.0000
Well-Being Scale	0.62	0.63	0.41	0.42
	*p* = 0.0000	*p* = 0.0000	*p* = 0.0000	*p* = 0.0000
Desired Changes Scale	−0.13	−0.08	−0.02	−0.10
	*p* = 0.002	*p* = 0.071	*p* = 0.609	*p* = 0.029
